# Astrocyte Diversity and Alcohol-Related Gliovascular Alterations in the Human Dorsal Striatum Revealed by Combined Morphometric and Ultrastructural Analyses

**DOI:** 10.3390/cells15100892

**Published:** 2026-05-14

**Authors:** Evalds Viguls, Anita Ilze Gulbe, Simons Svirskis, Valerija Groma, Sandra Skuja

**Affiliations:** 1 Division of the Biological Sciences, University of Chicago, 5841 S Maryland Avenue, Chicago, IL 60637, USA; 2 Joint Laboratory of Electron Microscopy, Rīga Stradiņš University, Kronvalda Blvd. 9, LV-1010 Riga, Latvia; 3 Institute of Microbiology and Virology, Rīga Stradiņš University, Ratsupites St. 5, LV-1067 Riga, Latvia

**Keywords:** astrocytes, heterogeneity, human striatum, alcohol exposure, Sholl analysis, GFAP immunohistochemistry, neurovascular interface

## Abstract

**Highlights:**

**What are the main findings?**

- Six recurrent astrocytic morphometric profiles were distinguished in the human striatum.
- Alcohol-exposed individuals showed increased white matter astrocyte density and gliovascular alterations.

**What are the implications of the main findings?**

- Sholl-based morphometrics support quantitative assessment of astrocyte morphology but highlight the need for 3D and multi-marker approaches to capture full astrocyte complexity.
- These findings support future studies of astrocyte–vascular remodeling in alcohol-related pathology using larger cohorts and integrated quantitative imaging to define functional outcomes.

**Abstract:**

Astrocytes are key regulators of neuronal, metabolic, and vascular homeostasis, yet their morphological diversity and involvement in alcohol-related brain pathology remain incompletely characterized. In this study, we investigated astrocytic morphology in the human striatum of control individuals and subjects with short- and long-term alcohol exposure using immunohistochemistry combined with Sholl-based morphometric analysis, and ultrastructural assessment. GFAP immunohistochemistry was used to identify astrocytes, assess their morphology, and manually quantify GFAP^+^ cells in gray and white matter, followed by Sholl-based morphometric analysis to characterize astrocytic branching architecture and spatial organization. The number of GFAP^+^ astrocytes differed between tissue compartments, with a significant increase in white matter in alcohol-exposed individuals and no detectable change in gray matter. Morphometric analysis revealed pronounced astrocytic heterogeneity across all study groups. Sholl-derived metrics supported the distinction of six recurrent astrocytic morphometric profiles in the human striatum, distinguished by soma size, branching complexity, process length, and cell territory size. These profiles were present across gray and white matter, indicating intrinsic astrocytic structural diversity. Ultrastructural analysis further revealed alcohol-associated alterations at the astrocyte–vascular interface, including swelling of perivascular astrocytic endfeet, accumulation of intermediate filaments, and focal reductions in vascular wall coverage. Together, these findings demonstrate substantial astrocytic structural diversity in the human striatum accompanied by alcohol-related gliovascular remodeling.

## 1. Introduction

Astrocytes are highly heterogeneous glial cells that differ in form and function across central nervous system (CNS) regions. Early neuroanatomists recognized structural diversity among astrocytes, distinguishing fibrous astrocytes in white matter (WM) from protoplasmic astrocytes in gray matter (GM), a foundational taxonomy described by Koelliker and later adopted by Ramón y Cajal [1,2]. More recent frameworks extend classical morphology to functional states, such as the putative neurotoxic A1 and neuroprotective A2 phenotypes [3], as well as multi-dimensional classifications that capture region- and species-specific nuances [4]. Recognition of distinct astrocytic phenotypes has opened new directions for research, focusing on the identification of reliable biomarkers and the development of targeted therapeutic strategies for neurodegenerative diseases, as these glial cells play critical roles in neuronal support, synaptic regulation, and the brain’s response to injury [5]. Verkhratsky and colleagues have further emphasized that astrocyte heterogeneity extends beyond traditional protoplasmic and fibrous types to include region-, function-, and species-specific variants [4]. Despite these advances, morphological categorization remains relatively underdeveloped. This is partly due to the remarkable plasticity of astrocytes in response to physiological and pathological stimuli, as well as the absence of universally accepted structural markers for consistent subtyping [5,6]. This heterogeneity is particularly important at the neurovascular interface, where astrocytes play a central role in maintaining cerebral homeostasis [7].

The blood–brain barrier (BBB) is one notable dynamic neurovascular interface involved in CNS homeostasis [8,9]. Disruption of BBB integrity can be exacerbated by chronic systemic stressors, including long-term alcohol consumption, which promotes neuroinflammation and cognitive dysfunction [10,11,12]. Given that the dorsal striatum is a subcortical input region of the basal ganglia and a key component of corticostriatal circuits implicated in reward learning, habit formation, and compulsive alcohol seeking, astrocytes at its synaptic and neurovascular interfaces may provide a cellular link between alcohol-associated vascular vulnerability, astrocytic structural heterogeneity, and gliovascular remodeling in alcohol use disorder [13,14,15,16]. Astrocytes are central to BBB structure and function, especially through their endfeet, which ensheath cerebral microvessels [17]. Several studies indicate that astrocytes exhibit regional variability and distinctive responses to pathological stimuli, suggesting that specific subsets may differentially influence BBB integrity [4,18]. Changes in astrocyte density, including increases observed in experimental models of chronic alcohol exposure, are likely to reflect reactive states associated with injury, inflammation, or synaptic dysfunction [19], and may shape neuroimmune signaling and microenvironmental regulation [20]. Nevertheless, the impact of chronic alcohol exposure on the structural interactions between vascular-associated astrocytes and the BBB in the human striatum remains poorly understood. Addressing this gap requires a detailed examination of astrocyte morphology and subcellular organization within the neurovascular unit.

Morphological alterations of astrocytes, including hypertrophy, loss of complexity, and ultrastructural organelle changes, are frequently observed in pathological conditions and are believed to influence functional outcomes [21,22]. Astrocytes exhibit diverse morphologies, ranging from the relatively simple, elongated processes of WM fibrous astrocytes to the highly branched architectures of GM protoplasmic astrocytes with fine peripheral processes [23,24,25]. The complex arbor of astrocytes, comprising multiple hierarchical branching orders that terminate in fine leaflets and endfeet, facilitates tight integration into the neurovascular unit [26,27]. In pathological states, astrocytes can develop swollen endfeet, reduced process complexity, and alterations in somatic morphology that weaken microvessel coverage and contribute to BBB disruption [28]. Alcohol exposure is particularly relevant in this context, as recent evidence indicates that astrocytes contribute to alcohol-related neuroinflammatory, metabolic, and structural brain alterations [11,12]. Thus, alcohol-associated pathology may involve not only changes in astrocyte number or glial fibrillary acidic protein (GFAP) expression, but also remodeling of astrocytic processes and perivascular compartments. To characterize such structural changes, studies commonly rely on established astrocytic markers that report cytoskeletal remodeling.

One commonly used marker is GFAP, an intermediate filament protein enriched in larger astrocytic processes. GFAP is widely used to identify astrocytic reactivity by immunohistochemistry (IHC), and its upregulation is supported by molecular and ultrastructural analyses [29,30]. Under physiological conditions, GFAP expression is higher in fibrous astrocytes of the WM than in protoplasmic astrocytes residing in the GM and is further increased upon reactive activation [29,31]. Moreover, structural signatures such as process hypertrophy and increased GFAP expression are often interpreted as reactive but may also reflect adaptive plasticity, whereas profound fragmentation and astrocytic atrophy are more indicative of degenerative astrocytic phenotypes [30,32]. In the context of the present study, GFAP-based immunolabeling provides a practical means to assess regional and alcohol-associated structural alterations in astrocytes, although it primarily captures the intermediate filament-rich cytoskeletal compartment rather than the full astrocytic territory. Thus, GFAP-based cytoskeletal labeling remains a sensitive tool for assessing regional and reactive structural changes, provided its compartment-specific limitations are considered.

Taken together, these considerations emphasize the need for an integrated structural analysis of astrocytes in the human striatum in the context of alcohol-associated pa-thology. Therefore, the present study had three related objectives: first, to quantify the density of GFAP-immunoreactive astrocytes in striatal GM and WM across control and alcohol-exposed groups; second, to determine whether recurrent astrocytic morphologies identified by GFAP immunolabeling at the light-microscopic level could be quantitatively supported by Sholl-derived morphometric descriptors. In this context, the identified profiles are interpreted as recurrent GFAP-based morphometric configurations that describe structural heterogeneity, without implying molecularly or functionally distinct astrocyte subtypes. The third objective was to assess alcohol-associated ultrastructural alterations at astrocyte–vascular and perisynaptic interfaces. Together, these approaches were intended to provide complementary structural perspectives on astrocytic organization rather than to establish direct ultrastructural correlates for individual morphometric profiles.

## 2. Materials and Methods

### 2.1. Tissue Samples

In the study, retrospectively archived human brain tissue samples from the dorsal striatum of 38 individuals were obtained from the Latvian State Center for Forensic Medical Examination (FMEC). The reported group sizes refer to unique individuals, with one formalin-fixed, paraffin-embedded (FFPE) striatal tissue specimen analyzed per subject. These specimens were collected during forensic autopsy post-mortem and preserved as FFPE tissue blocks according to the routine histopathology workflow, which was applied uniformly to control and alcohol-exposed specimens. Brain tissue samples were fixed in 10% neutral buffered formalin for at least 72 h prior to routine tissue processing. Following fixation, specimens were dehydrated through graded ethanol solutions, cleared in xylene, and embedded in paraffin. Serial sections of defined thickness were subsequently prepared from FFPE tissue blocks for further analysis. These procedures were applied uniformly to both alcohol-exposed and control groups to minimize methodological variability related to tissue processing. All histopathological, immunohistochemical, and electron microscopic analyses, as well as subsequent quantitative and morphological assessments, were conducted in full compliance with relevant local regulations and European Union requirements governing research involving human tissue.

To ensure donor anonymity, all cases were de-identified at FMEC prior to release, with each specimen assigned a unique laboratory code generated through a randomized process. Inclusion and exclusion criteria were applied during the collection phase. The presence or absence of alcohol-related pathology was determined through comprehensive forensic autopsy procedures, including both gross and microscopic examinations of multiple organs, such as the brain, liver, pancreas, lungs, and heart.

Alcohol-related conditions were identified by a board-certified forensic pathologist and supported by toxicological analyses, including quantitative assessment of blood ethanol concentrations. The post-mortem interval ranged from 7 to 37 h. Eligibility criteria and diagnostic categorization were aligned with the International Classification of Diseases, 10th Revision (ICD-10), with alcohol-related pathology defined based on documented autopsy findings and toxicological evidence.

All necessary ethical approvals were obtained prior to the commencement of the study. Conventional autopsies were conducted in accordance with the applicable legal and regulatory frameworks of the Republic of Latvia and the European Union, including the law enacted on 15 December 1992, On the Protection of the Body of Deceased Human Beings and the Use of Human Tissues and Organs in Medicine. The use of post-mortem human brain tissue for research purposes was approved by the Ethics Committee of RSU, in accordance with the principles of the Declaration of Helsinki. Ethics approval was initially granted on 17 December 2009, and subsequently renewed on 17 April 2025 (Decision No. 2-PĒK-4/666/2025). All research activities were conducted using fully anonymized material. No personal or identifying information was available to the investigators, ensuring full compliance with the General Data Protection Regulation and applicable ethical guidelines. Access to donor-related information was restricted exclusively to the forensic institution and was not available to the research team.

### 2.2. Experimental Design

A total of 38 human striatal tissue specimens were obtained from forensic autopsies and categorized into three groups based on comprehensive forensic case evaluations, including medical records, toxicological analyses, and documented history of alcohol use. The control group consisted of individuals with no evidence of alcohol abuse (*n* = 10, <37 years). The short-term alcohol use group (*n* = 12) comprised age-matched individuals with toxicological evidence of recent alcohol consumption but no indications of chronic abuse; this group was included specifically to disentangle the effects of acute alcohol exposure from those of prolonged use, independent of age-related factors. The long-term alcohol use group (*n* = 16) comprised individuals with documented chronic alcohol abuse, supported by medical and pathological findings, and included older individuals reflecting the cumulative impact of long-term alcohol consumption.

Astrocytes were quantified manually with counts performed both in the GM and WM. Initial qualitative assessment revealed pronounced morphological heterogeneity among astrocytes, characterized by the emergence of several visually distinct profiles. To substantiate these visual observations, Sholl analysis was applied, which confirmed structural differences in branching patterns and supported the recognition of recurrent profiles. Manual GFAP-positive (GFAP^+^) cell counts were performed across all cases, whereas Sholl-based morphometric analysis was performed on the selected subset of astrocytic profiles meeting predefined quality criteria; profile distribution remained descriptive. To complement light microscopic findings, ultrastructural examination was performed to assess astrocytic endfeet in close apposition to capillaries, thereby providing structural context for potential alterations in astrocyte–vascular interactions.

### 2.3. Immunohistochemistry

Astrocytes were identified by immunostaining for GFAP. A mouse monoclonal antibody against GFAP (clone ASTRO6, Invitrogen, Carlsbad, CA, USA, # PIMA512023) was applied at a dilution of 1:200. FFPE sections (4–5 μm) of the human striatum were deparaffinized, rehydrated, and subjected to antigen retrieval in 0.01 M citrate buffer (96 °C, 15 min). After blocking endogenous peroxidase activity with 3% hydrogen peroxide in methanol for 30 min, sections were incubated overnight at 4 °C with the primary antibody. Antigen–antibody complexes were visualized using the HiDef Detection™ HRP Polymer system (Cell Marque, Rocklin, CA, USA, # 954D-30) with diaminobenzidine (DAB, # 957D-30) as the chromogen. A brown coloration produced by the DAB chromogen was interpreted as a positive signal for GFAP immunoreactivity. Negative controls were processed using phosphate-buffered saline in place of the primary antibody. Sections were counterstained with Mayer’s hematoxylin, dehydrated, cleared, and mounted in Roti^®^ Histokitt (Carl Roth, Karlsruhe, Germany, # 6638.1). GM regions were identified based on their higher neuronal cell body density, neuropil-rich appearance, and relatively lower density of myelinated fiber bundles, whereas WM regions were delineated by the predominance of densely packed myelinated fiber tracts with sparse neuronal somata. The GM–WM boundaries were established through systematic microscopic evaluation of tissue architecture at low magnification prior to quantitative analysis. Field selection was subsequently performed only within clearly distinguishable compartments to ensure consistent regional sampling across all cases. GFAP expression was quantified by counting immunopositive astrocytes in 10 non-overlapping fields per sample at 400× magnification using a light microscope (Leica, Leitz BME, Wetzlar, Germany). Each analyzed field corresponded to approximately 0.159 mm^2^. Whole slide images were acquired using the Glissando Slide Scanner (Objective Imaging Ltd., Cambridge, UK). Quantification across the GM and WM was conducted using Aperio ImageScope software v12.2.2.5015 (Leica Biosystems, Buffalo Grove, IL, USA). To obtain a more comprehensive evaluation of GFAP expression across the entire tissue sample, astrocytes were counted in all 10 visual fields, and the counts were summed to yield a total GFAP^+^ cell number per case. Fields were selected systematically within predefined GM and WM regions to ensure representative sampling of the analyzed tissue compartment. To minimize selection bias, the entire region of interest was first systematically screened at low magnification, after which fields with intact tissue morphology, clearly identifiable astrocytic profiles, and adequate staining quality were selected for quantitative analysis. Areas affected by tissue folds, tears, edge artifacts, autolytic changes, or uneven staining were excluded from counting. This cumulative value was used for subsequent statistical analysis. IHC reactions were evaluated independently by two morphologists. Field selection and manual GFAP^+^ cell counting were performed in a blinded manner with respect to study group.

### 2.4. Sholl Analysis

In total, 205 astrocytes were screened from striatal tissue specimens to characterize the spectrum of astrocytic morphologies present in the samples. Sholl analysis was applied to determine whether the pronounced morphological differences in GFAP^+^ astrocytes observed during qualitative assessment could be supported by objective quantitative descriptors under standardized two-dimensional imaging conditions. This initial screening included astrocytes from the long-term alcohol user, short-term alcohol user, and control groups, and was performed using identical evaluation criteria across groups. Cells were included in the analysis only if they exhibited clearly identifiable soma and processes, adequate section preservation, and sufficient image sharpness. Cells affected by sectioning artifacts, incomplete labeling, or imaging limitations were excluded prior to further analysis. The remaining astrocytes were first assigned to six candidate GFAP-based profiles using qualitative light-microscopic screening criteria. These candidate profiles were then evaluated using Sholl-derived quantitative descriptors. Throughout the manuscript, the term “morphometric profile” refers to a recurrent GFAP-based astrocytic configuration that was initially recognized morphologically and subsequently supported by quantitative Sholl-derived descriptors. To ensure comparable representation across tissue compartments, an appropriate sampling strategy was applied, whereby an equal number of astrocytes was selected per profile from GM and WM in each study group. As a result of this sampling scheme, a total of 36 astrocytes meeting predefined quality criteria (well-preserved morphology and complete high-quality two-dimensional representations) were included in the quantitative Sholl analysis. This restrictive sampling strategy was intended to ensure comparable, high-quality two-dimensional astrocytic profiles for morphometric characterization, rather than to estimate the prevalence of each profile across study groups or tissue compartments.

Astrocyte images were extracted using Aperio ImageScope software v12.2.2.5015 (Leica Biosystems, Buffalo Grove, IL, USA). For each astrocyte, a region of interest with an initial size of 300 × 300 pixels was selected and uniformly enlarged to 400%, yielding a final image size of 1200 × 1200 pixels. Segmentation masks for Sholl analysis were generated from the extracted astrocyte images using FIJI/ImageJ (version 1.54f; National Institutes of Health, Bethesda, MD, USA), an open-source image analysis platform. GFAP^+^ structures were segmented using the Threshold function, followed by Watershed-based separation when necessary to improve the delineation of closely apposed signal. As the source material consisted of 4–5 µm sections, some astrocytic processes were only partially represented due to the two-dimensional sampling of a three-dimensional arbor. In such cases, minimal manual annotation was performed only to restore visually continuous GFAP^+^ branches that were clearly interrupted by sectioning-related concavity or signal discontinuity. No branches were added de novo.

For morphometric analysis, the soma and processes were separated prior to skeletonization. The soma mask was retained for soma size measurements but excluded from the Sholl pipeline in order to prevent artificial inflation of proximal intersections during skeletonization. The remaining GFAP^+^ processes were converted into binary masks, skeletonized, and analyzed using concentric Sholl rings centered on the soma. The soma center was selected manually for each astrocyte, and Sholl profiles were generated by counting intersections between the skeletonized astrocytic arbor and concentric circles at fixed radial increments. Radial increments for Sholl analysis were applied at constant 10-pixel intervals across all images. Pixel-to-micrometer conversion was performed using the calibrated image metadata and applied uniformly to all analyzed astrocytic profiles. For visualization, Sholl rings were overlaid on the corresponding processed astrocyte image, whereas intersection counts were derived exclusively from the skeletonized masks. The full image-processing workflow, including astrocyte selection, segmentation, soma removal, skeletonization, and Sholl ring overlay, is illustrated step-by-step in Supplementary Figure S1.

This standardized procedure ensured identical dimensions and magnification across all extracted astrocyte images, providing a consistent basis for subsequent mask generation and Sholl analysis. Accordingly, the generated masks should be interpreted as representations of GFAP^+^ astrocytic profiles visible in the section rather than complete three-dimensional astrocyte territories. Sholl analysis was used primarily to derive summary morphometrics of arbor architecture rather than to compare full intersection-by-radius profiles between groups. Metrics used to assess astrocyte morphology were adapted from prior studies [22,33]. To describe and compare morphometric profiles, we used six metrics: maximum process length, total number of intersections, the GFAP-labeled terminal-to-primary branch ratio, soma size, length-to-soma size ratio, and estimated cell territory size. For interpretability across metrics, each profile was additionally summarized as being above or below the global median. Maximum process length (µm) was defined as the straight-line distance from the soma outline to the most distal process reaching the maximum intersection radius; total number of intersections was defined as the total number of pixel-level crossings between skeletonized astrocytic processes and all concentric Sholl rings, summed across all radii, and used as a measure of arbor branching complexity; terminal-to-primary branch ratio was calculated as the number of terminal branch endings divided by the number of soma-originating primary processes; soma size (µm^2^) was quantified as the area occupied by the soma, obtained through segmentation of the soma outline; length-to-soma size ratio (µm^−1^) was calculated as maximum process length divided by soma area; estimated cell territory size (µm^2^) was derived from the last Sholl intersection radius, defined as the straight-line distance from the soma center to the most distal process intersection (last Sholl intersection), which represents the maximum width of the Sholl plot, and used to calculate the area enclosed by this radius.

### 2.5. Transmission Electron Microscopy

Transmission electron microscopy (TEM) was applied to assess the ultrastructure of the striatal brain samples. Sholl-based morphometry and TEM were treated as complementary analytical levels: the former quantified light-microscopic GFAP^+^ astrocytic profiles, whereas the latter evaluated alcohol-associated ultrastructural alterations at gliovascular and perisynaptic interfaces. TEM observations were therefore not assigned to individual GFAP-defined morphometric profiles. Specimens were fixed in 2.5% glutaraldehyde following standard laboratory procedures and subsequently postfixed in osmium tetroxide. After dehydration, the material was embedded in epoxy resin (Sigma-Aldrich, Buchs, Switzerland, # 45359). Semithin sections were prepared using an ultramicrotome (PowerTome, RMC Boeckeler, Boeckeler Instruments Pte Ltd., Singapore, Singapore), stained with 1% toluidine blue, and examined at 400× magnification using a light microscope (Leitz DMRB, Leica Microsystems, Wetzlar, Germany) for general structural evaluation. Ultrathin sections were then cut, mounted on formvar-coated nickel grids, and counterstained with 2% uranyl acetate followed by lead citrate. Imaging was performed on a JEM 1011 electron microscope (JEOL, Akishima, Tokyo, Japan). For ultrastructural assessment, up to ten transversely sectioned microvessels were randomly selected from each group and examined at a magnification of 6000–20,000×.

### 2.6. Statistical Analysis

Statistical computations were performed using GraphPad Prism version 9.0 (GraphPad Software Inc., San Diego, CA, USA). For IHC quantification, the individual subject was used as the statistical unit, with cumulative GFAP^+^ cell counts generated per case for the analyzed GM and WM compartments. Sholl-derived morphometric parameters were summarized using descriptive statistics and compared across the six morphometric profiles as an exploratory analysis. These comparisons were intended to characterize measurable morphometric differences between profiles rather than to provide a fully powered confirmatory classification. Robust nonparametric statistical methods with correction for multiple comparisons were applied to the IHC data to ensure the reliability of the findings. The distributional properties of continuous variables were assessed through complementary normality tests, specifically the D’Agostino-Pearson omnibus test and the Shapiro–Wilk test. Between-group comparisons were conducted using the non-parametric Kruskal–Wallis (KW) test for data violating normality assumptions. Post hoc pairwise comparisons employed the Benjamini, Krieger, and Yekutieli (BKY) false discovery rate correction procedure to control for type I error inflation inherent in multiple testing scenarios. Given that exhaustive pairwise comparisons across all six profiles and all morphometric parameters would have required a large number of low-powered tests, targeted post hoc comparisons were performed in an exploratory manner. For each morphometric parameter, comparisons were made against the profile whose median value was closest to the global median of that parameter, in order to identify profiles deviating most clearly from a central tendency within the dataset. Continuous variables were reported as median values with corresponding interquartile ranges (IQR) to provide robust measures of central tendency and dispersion. Statistical significance was established at an alpha level of *p* < 0.05 for all inferential analyses.

## 3. Results

### 3.1. Study Group Characteristics

The control group, consisting of young healthy individuals, had a median age of 30 years, with an IQR of 22–36 years and a range of 18–37 years. The age-matched short-term alcohol use group had a median age of 31 years (IQR, 28–33; range, 22–35 years). The long-term alcohol use group had a higher median age of 49 years (IQR, 45–56; range, 38–66 years).

Each of the first two groups included one female; the long-term group included four females, and the rest were males. The blood ethanol concentration expressed in per mille (‰) was from 0–1.48; 0–4.61, and 0–8.44, respectively.

### 3.2. Analysis of GFAP Expression

**Figure 1:**
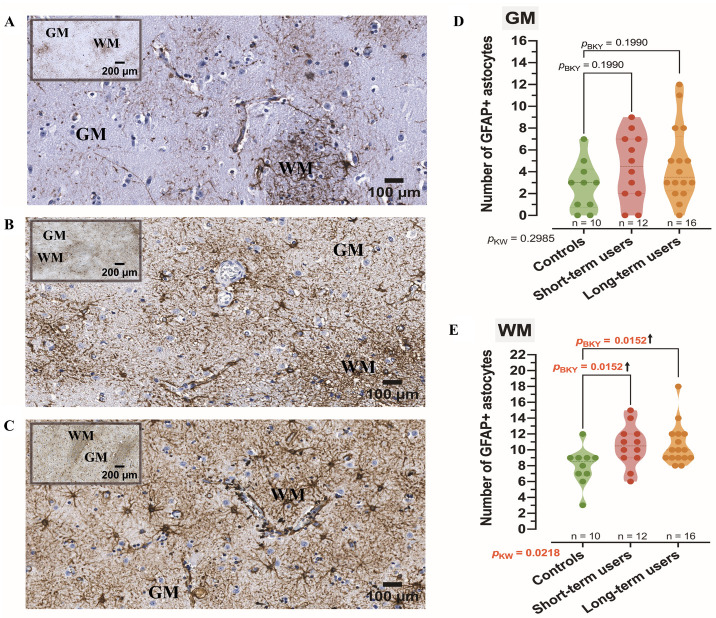
Representative immunostaining images and quantification of GFAP^+^ astrocytes in the striatal gray (GM) and white matter (WM) regions. (**A**) controls; (**B**) short-term alcohol users; (**C**) long-term alcohol users. Low-magnification insets in (**A**–**C**) indicate the corresponding GM and WM areas. WM was defined by the presence of myelinated fiber bundles and the absence of neuronal somata, whereas GM comprised the surrounding neuropil containing neuronal cell bodies. Violin plots depict the median number of GFAP^+^ astrocytes per field of view in the GM (**D**) and WM (**E**), reflecting overall abundance and typical field distribution across all study groups: control group (controls), short-term alcohol users (short-term users), and long-term alcohol users (long-term users). *p* values were derived: *P*_KW_ from the Kruskal–Wallis test; *P*_BKY_ from Benjamini, Krieger, and Yekutieli post-test. Significant *p* values are highlighted in red; upward arrows indicate an increase compared with controls.

Significantly more GFAP^+^ astrocytes were detected in the WM of both the short-term and long-term alcohol user groups compared to controls (Figure 1A–D). However, in the GM, no significant differences were found in the total number of GFAP^+^ astrocytes across study groups (Figure 1A–C,E). In all study groups, astrocytes exhibited morphological heterogeneity, including variations in soma size, the number and arborization pattern of processes.

**Figure 2:**
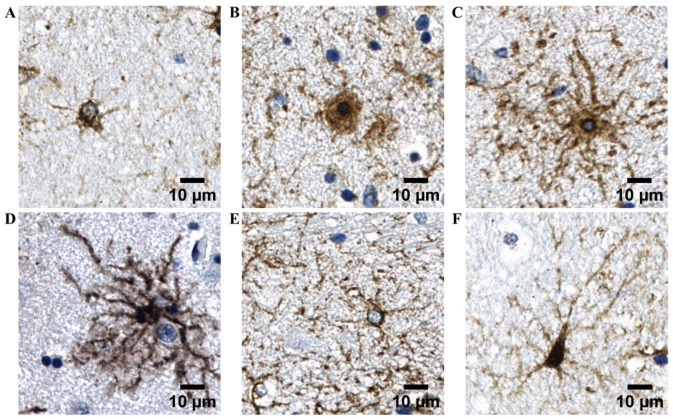
Representative images of GFAP^+^ astrocytes reflecting six recurrent astrocytic profiles (**A**–**F**) identified in the human striatum. Nuclei are counterstained with hematoxylin (blue), and GFAP immunoreactivity is visualized with DAB (brown).

IHC analysis did not reveal the presence of any GFAP-based morphometric profile specific to a particular study group. Although region-specific morphological tendencies were observed between astrocytes in the GM and WM (Figure 1D,E), these differences were primarily reflected in dominant structural features rather than the presence or absence of specific profiles. Astrocytes in the GM predominantly exhibited protoplasmic characteristics, whereas WM astrocytes more frequently displayed elongated somata and extended processes consistent with fibrous morphology. Importantly, all six GFAP-based morphometric profiles, initially identified according to qualitative morphological criteria and subsequently evaluated using Sholl-derived descriptors (Figure 2A–F), were detected in both GM and WM compartments, indicating that these profiles are not region-exclusive.

Morphometric profile 1. Astrocytes with below-median soma area and short, sparsely branched processes.

Morphometric profile 2. Astrocytes with enlarged, flattened somata and a small number of blunt processes.

Morphometric profile 3. Astrocytes with above-median soma area, long processes, and high branching complexity.

Morphometric profile 4. Astrocytes with below-median soma area and symmetrically arranged, highly branched processes.

Morphometric profile 5. Astrocytes with the smallest soma area and few processes of intermediate length.

Morphometric profile 6. Astrocytes with below-median soma area and comparatively long processes of variable length.

### 3.3. Sholl Analysis

#### 3.3.1. Morphological Stratification of Astrocytes into Six Morphometric Profiles

Sholl profiling, together with soma and territorial measurements, provided quantitative support for distinguishing six recurrent astrocytic morphometric profiles that corre-sponded to the profiles initially identified by qualitative IHC assessment (Figure 2A–F).

Global medians were as follows: maximum process length 33 µm (IQR, 22.86–36.78); number of total intersections across all rings 1783 (IQR, 910.25–3183); terminal-to-primary branch ratio 1.96 (IQR, 1.3–2.19); soma size 118.8 µm^2^ (IQR, 89.7–165.72); length-to-soma ratio 0.25 µm^−1^ (IQR, 0.13–0.37); and estimated cell territory size 253.5 µm^2^ (IQR, 209.46–424.22). For clarity, these values were used to classify each profile as above or below the global median across all metrics, as summarized in Table 1.

**Figure 3:**
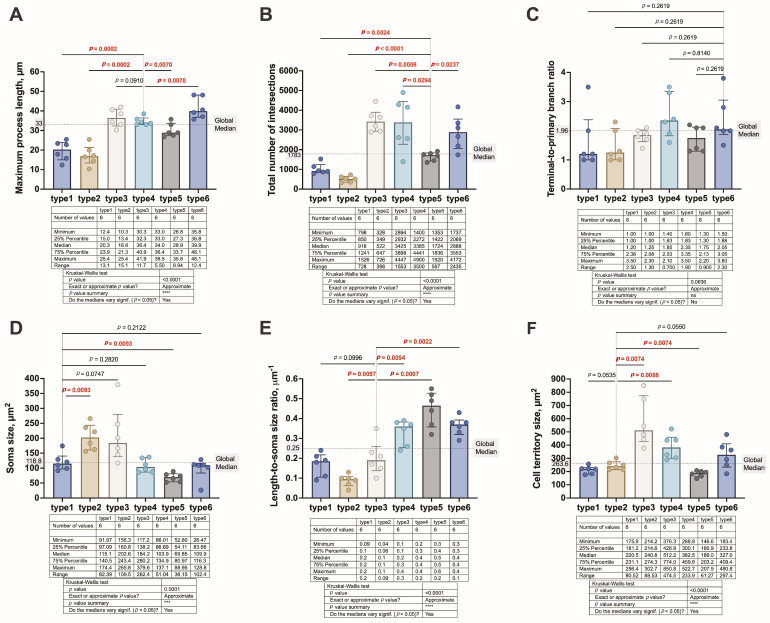
Astrocyte profiles differ in soma size and branching architecture. Morphometric parameters derived from Sholl analysis, along with soma and territory measurements, are shown for six astrocyte profiles, with data pooled across GM and WM. (**A**) Maximum process length; (**B**) total number of intersections; (**C**) terminal-to-primary branch ratio; (**D**) soma size; (**E**) length-to-soma size ratio; (**F**) estimated cell territory size. Horizontal dotted lines indicate global median values. Bars represent median values, with vertical lines denoting the interquartile range (25th to 75th percentiles). Individual observations are superimposed on each bar. The table beneath each panel displays median values with corresponding percentile ranges and *p*-values for overall between-group comparisons (Kruskal–Wallis test). Pairwise *p*-values reflect the significance levels derived from Benjamini–Krieger–Yekutieli post hoc analysis. For each parameter, comparisons are made against the profile whose median is closest to the global median. Significant *p* values are highlighted in red. Asterisks indicate the level of statistical significance: *** *p* < 0.001; **** *p* < 0.0001.

Across-identified profiles, five of the six metrics differed significantly: maximum process length, *p* < 0.0001, Figure 3A; total number of intersections, *p* < 0.0001, Figure 3B; soma size, *p* = 0.0001, Figure 3D; length-to-soma ratio, *p* < 0.0001, Figure 3E; and estimated cell territory size, *p* < 0.0001, Figure 3F, whereas the terminal-to-primary branch ratio showed only a trend toward significance, *p* = 0.0636, Figure 3C. Collectively, these results indicate that the identified morphometric profiles capture measurable differences in astrocyte size and branching architecture. Given that these parameters were derived from two-dimensional GFAP^+^ profiles in thin FFPE sections, they should be interpreted as relative descriptors of visible arbor architecture rather than unbiased measurements of complete three-dimensional astrocyte morphology.

#### 3.3.2. Profile-Specific Signatures

Profile 1 astrocytes showed an overall low-complexity morphometric profile, characterized by shorter maximal processes, with a median length of 20.3 µm, and fewer Sholl intersections, with a median of 918, both below the corresponding global medians. Their estimated territory size was also below the global median, with a median area of 220.5 µm^2^, and soma area remained slightly below the global median, with a median value of 115.1 µm^2^. Together, these features define a compact, low-arbor profile with limited radial extension.

Profile 2 astrocytes were distinguished primarily by an above-median soma area, with a median value of 202.6 µm^2^, combined with markedly reduced arbor complexity. These cells exhibited the lowest number of Sholl intersections among all profiles, with a median of 522, and shorter maximal processes, with a median length of 16.8 µm. The estimated territory size remained below the global median, with a median area of 240.8 µm^2^. Thus, profile 2 represents a soma-dominant morphometric profile, in which increased soma area is accompanied by limited process elaboration and restricted radial extension.

Profile 3 astrocytes combined an above-median soma area, displaying a median value of 184.2 µm^2^, with an above-median estimated territory size, featuring a median area of 512.2 µm^2^. These cells also showed high branching complexity, reflected by a median total intersection count of 3425, and an above-median maximal process length, with a median value of 36.4 µm. In contrast, the length-to-soma ratio remained below the global median, indicating that profile 3 is defined by increased absolute soma and arbor dimensions rather than by process extension relative to soma area.

Profile 4 astrocytes displayed a process-dominant architecture. Although their soma area was below the global median, with a median value of 103.9 µm^2^, these cells exhibited a high total number of Sholl intersections, with a median of 3385, above-median maximal process length, with a median value of 34 µm, and an above-median estimated territory size, with a median area of 382.5 µm^2^. Profile 4 also showed an above-median length-to-soma ratio, with a median value of 0.4 µm^−1^, consistent with a highly branched arbor extending from a below-median soma area. This profile therefore represents a high-complexity-per-soma morphometric profile.

Profile 5 astrocytes had the lowest median soma area among all profiles, with a median value of 69.85 µm^2^. This was accompanied by a below-median estimated territory size, with a median area of 189 µm^2^, and below-median branching complexity, reflected by a median intersection count of 1724 and a median maximal process length of 28.9 µm. However, the length-to-soma ratio was above the global median, with a median value of 0.5 µm^−1^. This indicates that, although profile 5 is compact and less branched overall, process extension is not proportionally reduced relative to soma area.

Profile 6 astrocytes exhibited a soma area below the global median, with a median value of 109.9 µm^2^, while showing an above-median arbor profile. This was characterized by high branching complexity, with a median intersection count of 2888, the highest median maximal process length among all profiles, with a median value of 39.9 µm, and an above-median estimated territory size, with a median area of 327 µm^2^. The length-to-soma ratio was also above the global median, with a median value of 0.4 µm^−1^. Thus, profile 6 represents a reach-oriented morphometric profile, defined by extended processes and above-median territory size in the context of below-median soma area. Although profiles 4 and 6 both occupy a process-dominant morphometric space, profile 4 is characterized by denser and more symmetric branching, whereas profile 6 is characterized by longer radial extension.

Overall, the six morphometric profiles occupied distinct regions of morphometric space defined by three main axes: (i) soma-dominant versus process-dominant architecture, (ii) compact versus expanded estimated territory, and (iii) lower versus higher branching complexity. The strongest discriminators across profiles were total number of intersections, estimated cell territory size, maximum process length, and length-to-soma ratio, all of which differed significantly across profiles. Soma area provided an additional discriminatory axis, separating the above-median soma profiles 2 and 3 from profiles with below-median soma area. Although the terminal-to-primary branch ratio did not reach significance across morphometric profiles in this dataset, its distribution was directionally consistent with the process-dominant profiles and may reflect higher within-profile variability or limited statistical power relative to the other parameters.

**Figure A1:**
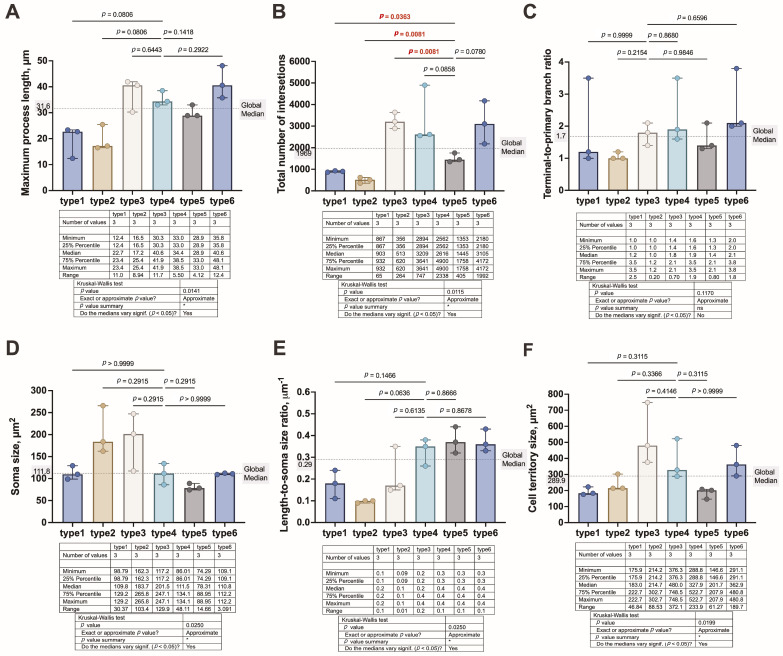
Astrocyte profiles in GM differ in soma size and branching architecture. Morphometric parameters derived from Sholl analysis, along with soma and territory measurements, are shown for six astrocyte profiles. (**A**) Maximum process length; (**B**) total number of intersections; (**C**) terminal-to-primary branch ratio; (**D**) soma size; (**E**) length-to-soma size ratio; (**F**) estimated cell territory size. Horizontal dotted lines indicate global median values. Bars represent median values, with vertical lines denoting the interquartile range (25th to 75th percentiles). Individual observations are superimposed on each bar. The table beneath each panel displays median values with corresponding percentile ranges and *p*-values for overall between-group comparisons (Kruskal–Wallis test). Pairwise *p*-values reflect the significance levels derived from Benjamini–Krieger–Yekutieli post hoc analysis. For each parameter, comparisons are made against the profile whose median is closest to the global median. Significant *p* values are highlighted in red. Asterisks indicate the level of statistical significance: * *p* < 0.1.

**Figure A2:**
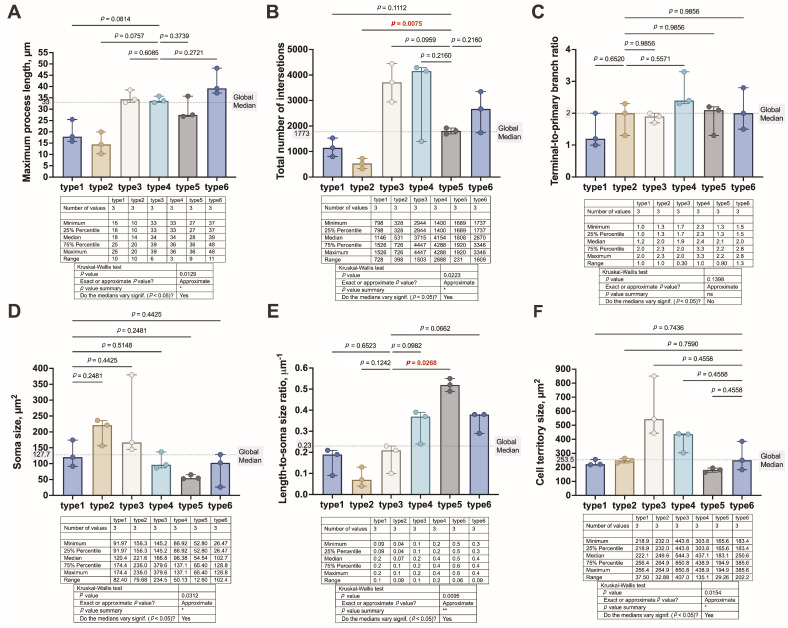
Astrocyte profiles in WM differ in soma size and branching architecture. Morphometric parameters derived from Sholl analysis, along with soma and territory measurements, are shown for six astrocyte profiles, with data pooled across WM. (**A**) Maximum process length; (**B**) total number of intersections; (**C**) terminal-to-primary branch ratio; (**D**) soma size; (**E**) length-to-soma size ratio; (**F**) estimated cell territory size. Horizontal dotted lines indicate global median values. Bars represent median values, with vertical lines denoting the interquartile range (25th to 75th percentiles). Individual observations are superimposed on each bar. The table beneath each panel displays median values with corresponding percentile ranges and *p*-values for overall between-group comparisons (Kruskal–Wallis test). Pairwise *p*-values reflect the significance levels derived from Benjamini–Krieger–Yekutieli post hoc analysis. For each parameter, comparisons are made against the profile whose median is closest to the global median. Significant *p* values are highlighted in red. Asterisks indicate the level of statistical significance: * *p* < 0.1; ** *p* < 0.01.

When astrocyte profiles were analyzed separately in GM and WM, most morphometric parameters remained broadly comparable between tissue compartments, with no consistent systematic differences across profiles (Figure A1 and Figure A2). This overall similarity suggests that the identified profiles are largely conserved across GM and WM compartments. Notable exceptions were observed in specific profiles. Profile 4 showed a pronounced GM–WM difference in the number of Sholl intersections, suggesting compartment-related variation in branching complexity. Profile 6 exhibited the largest divergence in estimated cell territory size, with WM astrocytes occupying a larger estimated territory size than their GM counterparts. In contrast, profile 5 displayed a visible compartment-dependent difference in the length-to-soma ratio. Aside from these profile-specific differences, other measured parameters showed only minor or negligible GM–WM variation, supporting the interpretation that tissue compartment may selectively modulate specific morphometric features rather than globally reshaping astrocyte architecture. Given the limited number of astrocytic profiles included in the compartment-specific Sholl comparisons, these GM–WM differences should be interpreted cautiously and considered exploratory.

### 3.4. Compartment-Specific Ultrastructural Alterations in Astrocytes

**Figure 4:**
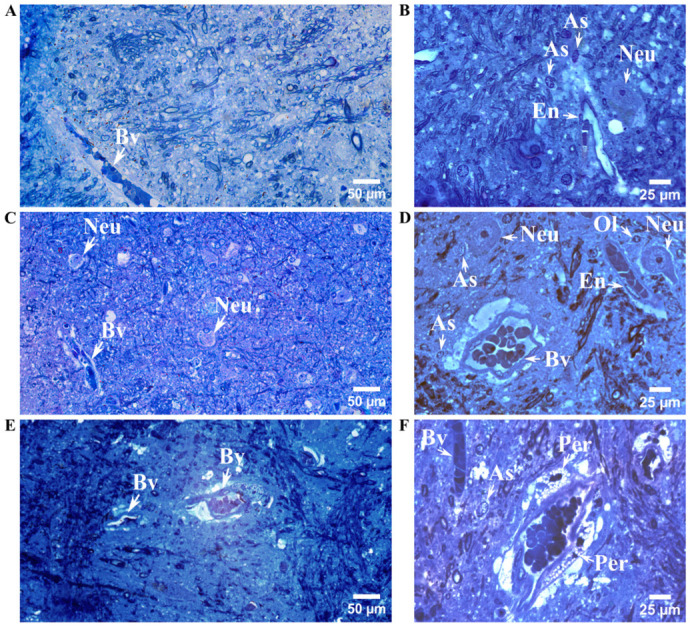
Representative light micrographs of toluidine blue-stained striatal sections obtained at 400× (**A**,**C**,**E**) and 600× (**B**,**D**,**F**) magnification in control (**A**,**B**), short-term alcohol use (**C**,**D**), and long-term alcohol use (**E**,**F**) groups. The striatum is organized into regions containing neuronal somata representing GM (**B**,**C**,**D**), interspersed with bundles of intensely blue-stained myelinated axons, visible in longitudinal or cross-sectional profiles, forming WM (**A**,**E**,**F**). Blood vessels (Bv) are distributed throughout the striatal region. Neurons (Neu) exhibit large euchromatic nuclei and prominent nucleoli, and lipofuscin granules are occasionally observed within their cytoplasm. Glial cells, including astrocytes (As) and oligodendrocytes (Ol), are characterized by smaller, more densely stained nuclei. The vascular wall contains endothelial cells (En) and pericytes (Per); in some pericytes, cytoplasmic lipofuscin (Li) inclusions are present.

Light microscopy of toluidine blue-stained semithin sections from the control group revealed the characteristic histological appearance of the striatal region. In GM, neurons displayed rounded cell bodies with scant cytoplasm and euchromatic nuclei, occasionally containing prominent nucleoli. Smaller nuclei with compact chromatin and minimal cytoplasm were also observed, corresponding to astrocytes, oligodendrocytes, or microglial cells. In the WM, both longitudinal and transverse profiles of myelinated axons of varying calibers were observed. Around smaller blood vessels, the perivascular space was inconspicuous, whereas larger vessels were surrounded by a thin, uniformly stained perivascular rim of even thickness (Figure 4A,B).

In the short-term alcohol user group, the overall histological organization of the striatum was largely comparable to that of controls, except for detectable vascular alterations (Figure 4C,D). In this group, the perivascular space displayed variable thickness, characterized by alternating regions of focal widening and marked narrowing. These alterations were most pronounced in the long-term alcohol user group, in which numerous round intracytoplasmic lipid inclusions were readily observed within vascular wall cells, even at the light microscopic level (Figure 4E,F).

**Figure 5:**
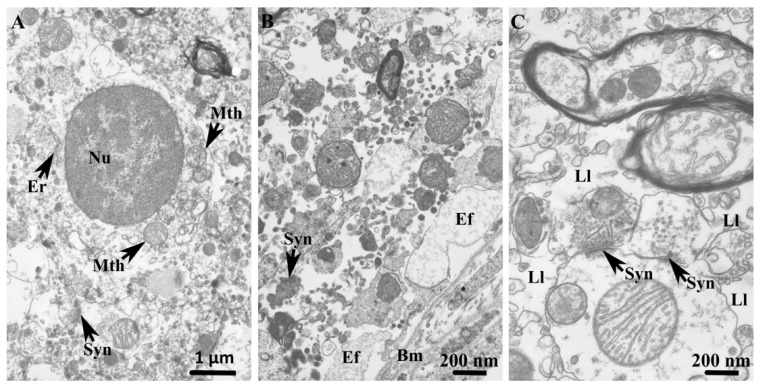
In the control group, transmission electron micrographs of the striatum reveal three ultrastructurally distinct compartments. (**A**) In the GM, the astrocytic soma displays a pale nucleus (Nu) containing finely dispersed chromatin, and is surrounded by an electron-lucent cytoplasm with sparse cisternae of rough endoplasmic reticulum (Er) and round mitochondria (Mth) of varying size. The astrocytic soma is in close proximity to synapses (Syn). (**B**) Astrocytic endfeet (Ef) extend longitudinally along the basal membrane (Bm) and exhibit variability in cytoplasmic organization. (**C**) In the neuropil, dendrite-formed synapses (Syn) are ensheathed by astrocytic leaflets (Ll) containing membrane-bound vesicles.

Transmission electron microscopy of control specimens revealed perivascular astrocytes with well-defined somata and preserved cytoplasmic ultrastructure (Figure 5A). The astrocytic cytoplasm exhibited low electron density and contained typical cytoplasmic organelles, as well as an oval to slightly irregularly contoured nucleus with a substantial proportion of euchromatin.

In control specimens, astrocytic endfeet closely ensheathed the neuropil-facing surface of the capillary basement membrane and contained mitochondria exhibiting heterogeneous morphologies, along with variably developed vacuoles and enlarged cisternae of the endoplasmic reticulum (Figure 5B). The capillary basement membrane was of relatively uniform thickness, with occasional focal duplications encircling pericytic profiles. Astrocytic endfoot coverage of the basement membrane was variable and predominantly thin, with localized areas of expansion. Filaments within astrocytic terminal processes were rare and sparsely distributed.

Within the surrounding neuropil, synapses were frequently ensheathed by astrocytic processes (Figure 5C). Perisynaptic astrocytic leaflet processes were often closely associated with asymmetric synapses, which were distinguished by a thickened postsynaptic membrane and round synaptic vesicles in the presynaptic pole. These leaflet processes exhibited an electron-lucent cytoplasm containing sparse mitochondria and variably sized membrane-bound vesicles. Intercellular junctions between adjacent astrocytic processes were commonly observed, consistent with preserved astrocytic coupling.

**Figure 6:**
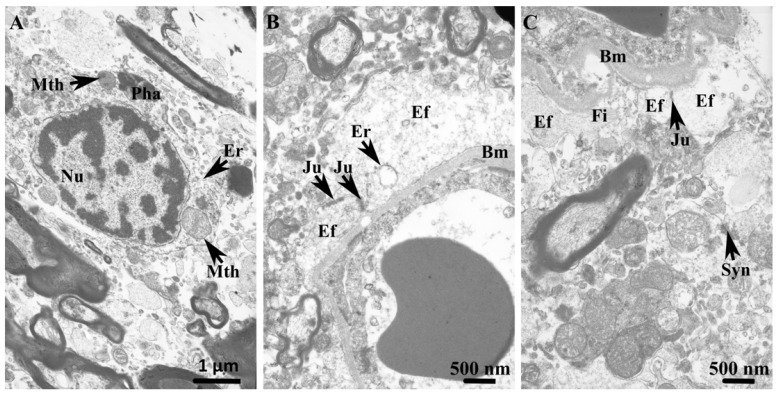
In the short-term alcohol user group, transmission electron micrographs of the striatum highlight ultrastructural alterations in astrocytic somatic and perivascular endfoot compartments. (**A**) In the WM, the astrocytic soma exhibits a nucleus (Nu) with chromatin forming small and large clumps, particularly along the nuclear membrane. The cytoplasm contains cisternae of the endoplasmic reticulum (Er), free ribosomes, and round mitochondria (Mth) of variable size, and occasional phagosomes (Pha). (**B**) Near the basal membrane (Bm), astrocytic endfeet (Ef) of varying size with heterogeneous cytoplasmic content are present. Neighboring astrocytic endfeet are in close apposition, forming multiple intercellular junctions (Ju). (**C**) Endfeet (Ef), exhibiting an electron-lucent cytoplasm and containing cytoplasmic filaments (Fi), are observed with preserved intercellular junctions between them. Synaptic profiles (Syn) are also evident within the surrounding neuropil.

In the short-term alcohol user group, astrocytic cell bodies exhibited centrally positioned nuclei characterized by a reduced proportion of euchromatin. The cytoplasm contained mitochondria, endoplasmic reticulum, and other common cytoplasmic organelles. In contrast, astrocytic endfeet opposed to the neuropil-facing surface of the capillary basement membrane were often depleted of cytoplasmic organelles and showed prominent accumulations of filaments adjacent to the perivascular interface (Figure 6A,B). The capillary basement membrane itself did not display overt alterations in thickness or continuity. Within the surrounding neuropil, small, myelinated axons lacked round or oval profiles characteristic of control specimens, but instead frequently exhibited highly irregular and convoluted outer contours. Between these axons, fragmented astrocytic processes were commonly observed, containing filaments with haphazard orientation. Enlarged and elongated mitochondria were frequently identified within these astrocytic processes. In addition, neurons were occasionally observed in close apposition to the capillary wall, leaving a narrow perivascular space for astrocytic coverage (Figure 6C).

**Figure 7:**
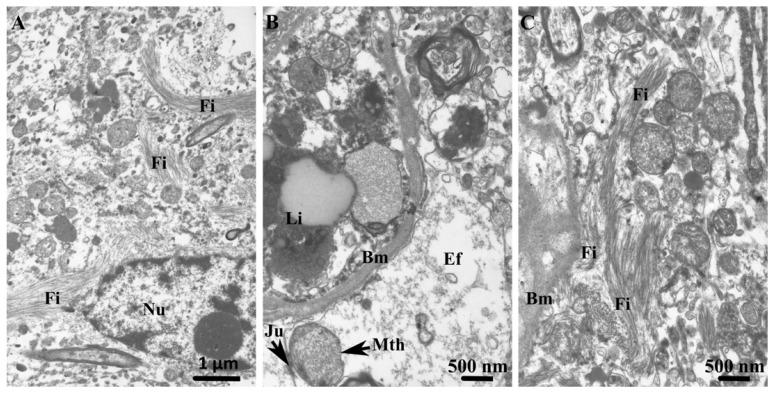
In the long-term alcohol user group, transmission electron micrographs of the striatum highlight ultrastructural alterations in astrocytic somatic and endfoot compartments. (**A**) In the GM, the astrocyte displays an oval, a pale nucleus (Nu) containing small amounts of irregularly distributed heterochromatin, predominantly along the nuclear envelope; a prominent nucleolus is present. The astrocytic soma is densely packed with filaments (Fi), and fragments of astrocytic processes extend in multiple directions and are likewise enriched in filamentous structures. (**B**) Adjacent to the basal membrane (Bm), a swollen astrocytic endfoot (Ef) containing enlarged mitochondria (Mth) is present. Intercellular junctions (Ju) are observed between neighboring astrocytic endfeet. Lipofuscin (Li) is present in the pericyte on the luminal side of the capillary. (**C**) An astrocytic endfoot adjacent to the basal membrane (Bm) exhibits a markedly reduced cytoplasmic matrix, with its interior almost entirely filled with densely packed filaments (Fi).

In the long-term alcohol user group, astrocytic nuclei exhibited a balanced distribution of euchromatin and heterochromatin, with small heterochromatin clumps evenly dispersed throughout the nucleoplasm (Figure 7A). The perinuclear cytoplasm contained identifiable organelles, including mitochondria and dilated cisternae of rough endoplasmic reticulum, and cell junctions between neighboring astrocytes were preserved. At the capillary interface, the neuropil-facing portion of the capillary basement membrane was focally thickened. Perivascular astrocytic endfeet were frequently swollen and contained cellular debris as well as enlarged mitochondria (Figure 7B). A high density of cytoplasmic filaments was observed in these astrocytic terminals (Figure 7C). Pericytes adjacent to the basement membrane exhibited numerous lysosomes (Figure 7B). Within the surrounding neuropil, elongated astrocytic processes in close proximity to neuronal somata—often enriched with lipofuscin inclusions—displayed an electron-lucent cytoplasm with only sparse organelles, including small, oval mitochondria. Synaptic regions were ensheathed by very thin and structurally depleted astrocytic leaflets.

Together with the GFAP-based morphometric findings, these ultrastructural observations indicate alcohol-associated astrocytic remodeling at gliovascular and perisynaptic interfaces, although the TEM findings were not assigned to individual morphometric profiles.

## 4. Discussion

In this study, we used two complementary structural approaches to examine astrocytic organization in the human striatum. GFAP-based light microscopy combined with Sholl-derived morphometry was used to identify and quantify six recurrent GFAP^+^ astrocytic morphometric profiles at the light-microscopic level. In parallel, transmission electron microscopy was used to assess alcohol-associated ultrastructural alterations at astrocyte–vascular and astrocyte–synaptic interfaces, particularly in individuals with long-term alcohol exposure. Traditional IHC-based approaches to astrocyte characterization rely largely on cell density, relative optical density, or qualitative morphological descriptors [34]. Although these methods are valuable, they provide limited insight into territorial organization and arbor architecture. By applying Sholl analysis to selected GFAP^+^ astrocytes, we extended standard IHC observations with quantitative measures of branching complexity, process length, and cell territory size. This combined approach showed that astrocytic heterogeneity in the human striatum is not only visually apparent but also supported by measurable morphometric differences. The conceptual value of this classification lies in providing a quantitative morphometric framework for describing recurrent GFAP-based structural configurations in the human striatum, while remaining distinct from classifications based on discrete molecular phenotypes or profile-specific alcohol effects. At the level of GFAP^+^ astrocyte counts, we observed a significant increase in the number of GFAP^+^ astrocytes in the WM of both short- and long-term alcohol users, whereas no group differences were detected in GM, indicating a compartment-specific astrocytic alteration associated with alcohol exposure. This pattern is consistent with growing evidence that astrocyte reactivity in alcohol use disorder is preferentially associated with WM pathology, where astrocytes are critically involved in axonal support, metabolic coupling, and myelin integrity [12,28]. In contrast, the absence of group differences in gray matter GFAP^+^ cell numbers suggests that astrocytic alterations in these regions may primarily manifest as morphological remodeling rather than changes in astrocyte number, in line with recent reviews emphasizing that astrocyte reactivity encompasses diverse structural and phenotypic states not necessarily captured by GFAP-based cell counts alone [11,30].

Previously, Oberheim et al. reported four distinct GFAP^+^ astrocyte subtypes distributed across the GM and WM of the human neocortex, underscoring the remarkable morphological heterogeneity present within a single region of the central nervous system [35]. Consistent with these observations, our findings indicate that the human striatum exhibits considerable morphological heterogeneity, with evidence for six recurrent astrocytic profiles. In line with the conceptual framework proposed by Verkhratsky et al., astrocytic heterogeneity may arise within the same condition or emerge at different stages of disease progression [4]. In the present study, morphometric heterogeneity was observed across all study groups. However, as our classification is based on GFAP morphology alone, these profiles should not be interpreted as definitively stable astrocyte subtypes. They may reflect stable subpopulations, dynamic reactive states, or a combination of both. This interpretation is consistent with recent studies showing that astrocyte heterogeneity is region-specific and multidimensional, involving morphological, molecular, and disease-associated components [4,22,36]. In this context, the present GFAP-based profiles should be regarded as morphometric configurations rather than discrete molecular or functional astrocyte subtypes.

Other studies have shown that, despite similar somatic volumes and numbers of primary branches, striatal astrocytes occupy significantly larger territories than hippocampal astrocytes, resulting in coverage of a greater number of neuronal somata but fewer excitatory synapses per astrocytic area [37]. Based on our findings, the presence of striatal astrocyte profiles characterized by either elongated processes or, conversely, markedly shorter processes suggests that territorial dimensions within the striatum may vary rather than be uniform. Such variability may influence local neuron–glia interactions and could contribute to region-specific differences in astrocytic support functions.

It has been reported that protoplasmic astrocytes typically extend 5 to 10 primary processes from the soma, which further branch into thinner processes, leaflets, and endfeet [38]. Within the constraints of two-dimensional GFAP-based profiling, our Sholl-based morphometric analysis provides quantitative support for this interpretation by demonstrating that astrocyte profiles differ substantially in branching architecture, as reflected by the number of Sholl intersections and maximal process length. This is consistent with previous GFAP-based morphometric work showing that Sholl-derived and related branching parameters can reveal treatment- or injury-associated astrocytic structural remodeling [39]. Profiles characterized by reduced numbers of intersections and shorter maximal radii represent low-complexity arbors with limited spatial reach, whereas other profiles exhibit extensive branching with high intersection counts and extended radial expansion. Together, these differences suggest that visible GFAP^+^ arbor architecture is not uniform within the striatum but can be organized into recurrent structural profiles with differing estimated spatial reach and branching complexity. From a functional perspective, profiles with longer processes, larger estimated territories, and greater branching complexity may reflect configurations with broader spatial extension within the surrounding neuropil and, where processes are associated with vascular elements, more extensive gliovascular interfaces. In contrast, compact or soma-dominant profiles may represent more spatially restricted configurations. Although these functional implications were not directly assessed in the present study, such morphometric differences are consistent with the recognized importance of astrocyte morphology for local synaptic support, perivascular homeostasis, and neurovascular unit organization [22,27,40].

To our knowledge, there are no published quantitative morphometric measurements of individual astrocyte territorial size in the adult human striatum. Our Sholl-based analysis thus provides an estimate of astrocyte spatial extent in this region and enables comparison with experimental measurements from animal studies. In the mouse dorsolateral striatum, individual astrocytes occupy territories of approximately 2338 ± 110 µm^2^ based on membrane-targeted fluorescent reporters that enable visualization of full astrocyte territories, which translates to an effective diameter of roughly 55–60 µm assuming a circular projection [41]. Bushong and colleagues demonstrated that GFAP visualizes only ~15% of the total astrocyte volume in hippocampal tissue, implying that GFAP-based measures systematically underestimate true domain size [42]. Taking this limitation into account, our measured GM and WM astrocyte diameters (~71 µm and ~67 µm, respectively) may represent only partial domain dimensions. When considered in this context, the estimated full-domain size falls within the range reported in previous studies [35].

Beyond estimated territory size, Sholl analysis revealed that multiple independent morphometric parameters contribute to astrocyte stratification, including total intersection count, maximum process length, and the length-to-soma size ratio. Notably, these parameters were among the strongest discriminators between profiles, indicating that astrocytic heterogeneity in the striatum cannot be adequately described by soma size alone. Instead, the relative balance between soma dimensions and arbor elaboration emerges as a defining feature, separating soma-dominant morphometric profiles from process-dominant profiles. These distinctions may influence the extent to which astrocytes sample local synaptic, metabolic, and vascular microenvironments, and may therefore be relevant to differences in astrocyte participation in neurovascular unit organization and tissue homeostasis.

While ultrastructural three-dimensional reconstructions by Mathiisen et al. demonstrated that astrocytic endfeet interdigitate to provide continuous and complete coverage of the abluminal endothelial surface in the hippocampal stratum moleculare of CA1 in mice [43], ultrastructural analysis of the human striatum revealed focal vascular wall regions in alcohol user groups in which the endothelial surface was not fully ensheathed by astrocytic endfeet, potentially suggesting altered astrocyte-mediated vascular support. Mathiisen et al. further reported that such uncovered vascular domains could be contacted by microglial processes; however, no microglial involvement was detected in these regions in the alcohol user groups. Notably, large-scale imaging data from the UK Biobank demonstrate that even moderate alcohol consumption is associated with reduced striatal volume and microstructural alterations in the WM, with stronger effects observed at higher levels of alcohol intake [44]. Taken together, these observations suggest that astrocytic morphological remodeling in alcohol-exposed individuals may be consistent with macroscopic changes in brain volume and connectivity detected in large-scale neuroimaging studies.

Hösli et al. demonstrated that nearly all cortical GM astrocytes are found in close contact with at least one blood vessel, with deeper cortical layers showing increased contact density per astrocyte [45]. Lorin et al., analyzing both the mouse hippocampus and cortex, similarly reported that most astrocytes are arranged around three blood vessels, with some extending contacts to as many as seven [27]. Collectively, these studies support the idea that cortical and hippocampal astrocytes form an extensive and widespread gliovascular interface. Gliovascular contacts were not systematically quantified in the present study; however, ultrastructural analysis revealed focal regions in the long-term alcohol user group in which astrocytic endfoot apposition to the vascular wall appeared reduced. Although functional consequences were not directly assessed, swelling of astrocytic endfeet, filament accumulation, and incomplete vascular coverage may indicate altered astrocyte-mediated perivascular support of BBB maintenance and neurovascular coupling. Similarly, thinning or structural depletion of perisynaptic astrocytic leaflets may reflect altered astrocyte–synapse structural support within the striatum. In the absence of direct measurements of BBB permeability and neurovascular function, these observations should be interpreted as structural indicators of gliovascular remodeling rather than as direct evidence of BBB breakdown.

Astrocytic endfeet are highly specialized structures, and are densely packed with mitochondria, cisternae of rough endoplasmic reticulum, and vesicles, reflecting their complex role in neurovascular interactions. They also contain protein translation machinery, metabolic enzymes, adhesion proteins, and scaffold proteins that interact with plasma membrane proteins, including channels, transporters, and receptors—essential for astrocyte–vasculature communication [40]. Göbel et al. demonstrated that a cortical stab-wound injury and BBB disruption trigger the formation of a prominent mitochondrial-enriched compartment in astrocytic endfeet, enabling vascular remodeling through fusion-regulated clustering [46]. Our ultrastructural analysis performed using specimens from long-term alcohol-exposed individuals revealed similar mitochondrial accumulations within astrocytic endfeet, suggesting that mitochondrial redistribution within astrocytic endfeet may represent a shared feature of astrocytic remodeling under conditions involving vascular stress. In addition to mitochondrial reactivity, our TEM analysis revealed progressive-like changes in the filament content of astrocytic endfeet. In control samples, astrocytic endfeet adjacent to the capillary basement membrane contained only sparse filaments. These were more frequently observed in the short-term alcohol user group and were a common feature in the long-term alcohol-exposed group. Increased filament accumulation within astrocytic endfeet may reflect a shift toward a more structurally reinforced but less functionally specialized endfoot adaptation, which may be associated with altered structural organization at the gliovascular interface. This interpretation is consistent with experimental data showing that chronic high-dose ethanol exposure increases GFAP expression and impairs glymphatic function—a brain-wide waste clearance pathway [47]. On the one hand, GFAP-based morphometry demonstrated altered branching complexity and territory size of parenchymal astrocytes in the alcohol group. On the other hand, our ultrastructural analyses revealed significant changes in perivascular astrocytic endfeet and BBB morphology in the same samples. Although these two analytical levels target distinct astrocytic compartments, the GFAP-rich parenchymal cytoskeleton versus the GFAP-poor vascular endfeet, the parallel alterations observed at these two structural levels suggest that alcohol-associated astrocytic changes were detectable in both parenchymal and perivascular compartments.

However, existing astrocyte classification schemes remain largely descriptive and are primarily based on histological observations, limiting their ability to capture the full spectrum of regional and functional heterogeneity across the CNS. A comprehensive and systematic morphological analysis of astrocytes across both GM and WM, ideally integrated with modern approaches such as three-dimensional reconstruction and single-cell transcriptomics, is still lacking. Recent single-cell RNA sequencing studies have demonstrated that while some astrocyte populations share gene expression profiles across regions, others exhibit region-specific signatures, particularly within WM [36]. Establishing how such molecular heterogeneity relates to regional morphology and pathology will be an important goal for future studies.

Previous studies have also highlighted methodological limitations in the quantitative assessment of GFAP^+^ astrocytes in FFPE tissue and emphasized the inherent subjectivity of morphological evaluation, even when reproducible scoring systems are applied [48]. Accordingly, we cannot exclude that the profiles identified in the present study may partly reflect observer-dependent bias, underscoring the need for validation in larger and more diverse cohorts.

### Limitations and Future Directions

Several limitations of the present study should be acknowledged. First, the Sholl-based quantitative analysis was performed on a relatively small subset of astrocytes selected according to strict quality criteria. Accordingly, the resulting morphometric profiles should be interpreted as exploratory structural configurations rather than as estimates of profile prevalence or definitive astrocyte subtypes. Second, astrocytic morphometric profiles were defined using GFAP immunolabeling and two-dimensional light microscopy, which capture the primary cytoskeletal framework rather than the full distal arborization or complete territorial domains [49]. Because the analysis was performed on thin 4–5 µm tissue sections, Sholl-derived parameters, including total number of intersections, maximum process length, and estimated territory size, reflect visible GFAP^+^ profiles within the section rather than complete three-dimensional astrocyte arbors. These measures may therefore be influenced by section plane, soma position within the section, process orientation, and incomplete arbor representation.

Third, the long-term alcohol use group was older than the control and short-term alcohol use groups, and age-related astrocytic changes therefore represent a potential confounding factor that cannot be fully separated from long-term alcohol-associated pathology in this group. The presence of increased GFAP^+^ astrocyte numbers in the age-matched short-term alcohol use group suggests that the WM finding is unlikely to be explained by aging alone; however, long-term alcohol exposure, age, and alcohol-related systemic pathology may have combined effects that should be disentangled in larger age-matched cohorts.

Additional limitations should also be considered. The present classification was based on GFAP-defined morphology alone; therefore, the identified morphometric pro-files should not be considered directly equivalent to established astrocyte classifications such as protoplasmic/fibrous or A1/A2 phenotypes, which require additional molecular and functional criteria. Although consistent classification criteria were applied across all samples, some degree of observer-dependent bias cannot be excluded. In addition, the retrospective forensic nature of the material limited the availability of standardized clinical metadata, including lifetime alcohol consumption patterns, treatment history, comorbidities, and agonal state. While the postmortem interval ranged from 7 to 37 h and all samples were processed using uniform fixation protocols, potential effects of postmortem delay and agonal factors on GFAP immunoreactivity and astrocyte morphology cannot be fully excluded. Finally, given the known anatomical and functional heterogeneity of the striatum, the lack of precise subregional differentiation, such as caudate nucleus versus putamen, may have obscured region-specific effects.

Quantitative assessment of morphometric profile abundance was beyond the scope of the present study. Accordingly, the present data do not determine whether alcohol-associated pathology selectively affects specific morphometric profiles. Future studies using additional astrocytic markers such as ALDH1L1 and S100B, together with thicker sections, confocal z-stack imaging, three-dimensional reconstruction, marker-based phenotyping, and single-cell or spatial transcriptomic approaches, will be important for validating the present findings and for determining how visible GFAP^+^ profiles relate to complete astrocyte territories and alcohol-associated astrocytic remodeling.

## 5. Conclusions

This study provides evidence that astrocytic heterogeneity in the human dorsal striatum is substantial, quantitatively definable, and observed across GM and WM, as well as across control and alcohol-exposed individuals. Using GFAP-based immunohistochemistry combined with Sholl-derived morphometric analysis, we identified six recurrent astrocytic morphometric profiles that differ significantly in soma dimensions, arbor complexity, process length, and territorial extent, indicating a range of morphological variations in both healthy and pathological conditions. These profiles represent recurrent GFAP-based morphometric configurations rather than discrete biological subtypes or direct functional identities.

Alcohol exposure was associated with a selective increase in the number of GFAP^+^ astrocytes in WM, whereas no corresponding alterations were observed in GM, indicating regionally distinct astrocytic responses within the striatum. Morphometric findings additionally suggest that astrocytic alterations in alcohol-exposed individuals may include structural reorganization alongside alterations in cell numbers. However, determining which morphometric profiles are selectively affected by alcohol exposure would require quantitative assessment of astrocyte numbers within each profile relative to controls.

Ultrastructural analysis further revealed alterations of the gliovascular interface in long-term alcohol-exposed individuals, characterized by astrocytic endfoot swelling, filament accumulation, and incomplete coverage of the capillary basement membrane. These findings suggest that alcohol-associated astrocytic pathology may involve the perivascular niche in addition to the parenchymal GFAP-labeled compartment.

Collectively, our findings point to astrocytes as important contributors to alcohol-related striatal pathology and emphasize the value of integrating quantitative morphometrics with ultrastructural analysis to capture complementary aspects of astrocytic organization in the human striatum.

## Figures and Tables

**Table 1:** Summary of astrocyte profile metrics relative to the global median.

Morphometric Profile	Maximum Process Length, µm	Total Number of Intersections	Terminal-to-Primary Branch Ratio	Soma Size, µm^2^	Estimated Cell Territory Size, µm^2^	Length to Soma Size Ratio, µm^−1^
1	<33	<1783	<1.96	<118.8	<253.5	<0.25
2	<33	<1783	<1.96	>118.8	<253.5	<0.25
3	>33	>1783	<1.96	>118.8	>253.5	<0.25
4	>33	>1783	>1.96	<118.8	>253.5	>0.25
5	<33	<1783	<1.96	<118.8	<253.5	>0.25
6	>33	>1783	>1.96	<118.8	>253.5	>0.25

## Data Availability

All data used in this article are included in the article. Additional data and materials are available upon request from the corresponding author.

## Supplementary Materials

The following supporting information can be downloaded at: https://www.mdpi.com/article/10.3390/cells15100892/s1, Figure S1: Workflow for astrocyte segmentation and Sholl analysis. (A) Original GFAP-immunostained astrocyte image extracted from a whole-slide image, original magnification ×400. (B) Binary mask generated in FIJI/ImageJ using intensity thresholding and watershed-based separation. (C) Soma mask used for soma size measurement. (D) GFAP-positive process mask after removal of the soma from the Sholl pipeline. (E) Processed binary input used for morphometric analysis. (F) Skeletonized representation of the astrocytic arbor used for intersection quantification. (G) Concentric Sholl rings overlaid for visualization; intersection counts were calculated on the skeletonized image rather than on the overlaid image.

## Abbreviations

The following abbreviations are used in this manuscript:

GFAP	Glial fibrillary acidic protein
CNS	Central nervous system
BBB	Blood–brain barrier
IHC	Immunohistochemistry
GM	Gray matter
WM	White matter
TEM	Transmission electron microscopy
IQR	Interquartile range
