# Supplementary material for: Astrocyte Diversity and Alcohol-Related Gliovascular Alterations in the Human Dorsal Striatum Revealed by Combined Morphometric and Ultrastructural Analyses
Source: Cells. 2026 May 14;15(10):892. doi: 10.3390/cells15100892 (PMC13204211; doi:10.3390/cells15100892)
Supplement: Supplementary file 1 [file cells-15-00892-s001.zip › cells-4296363-supplementary.pdf]

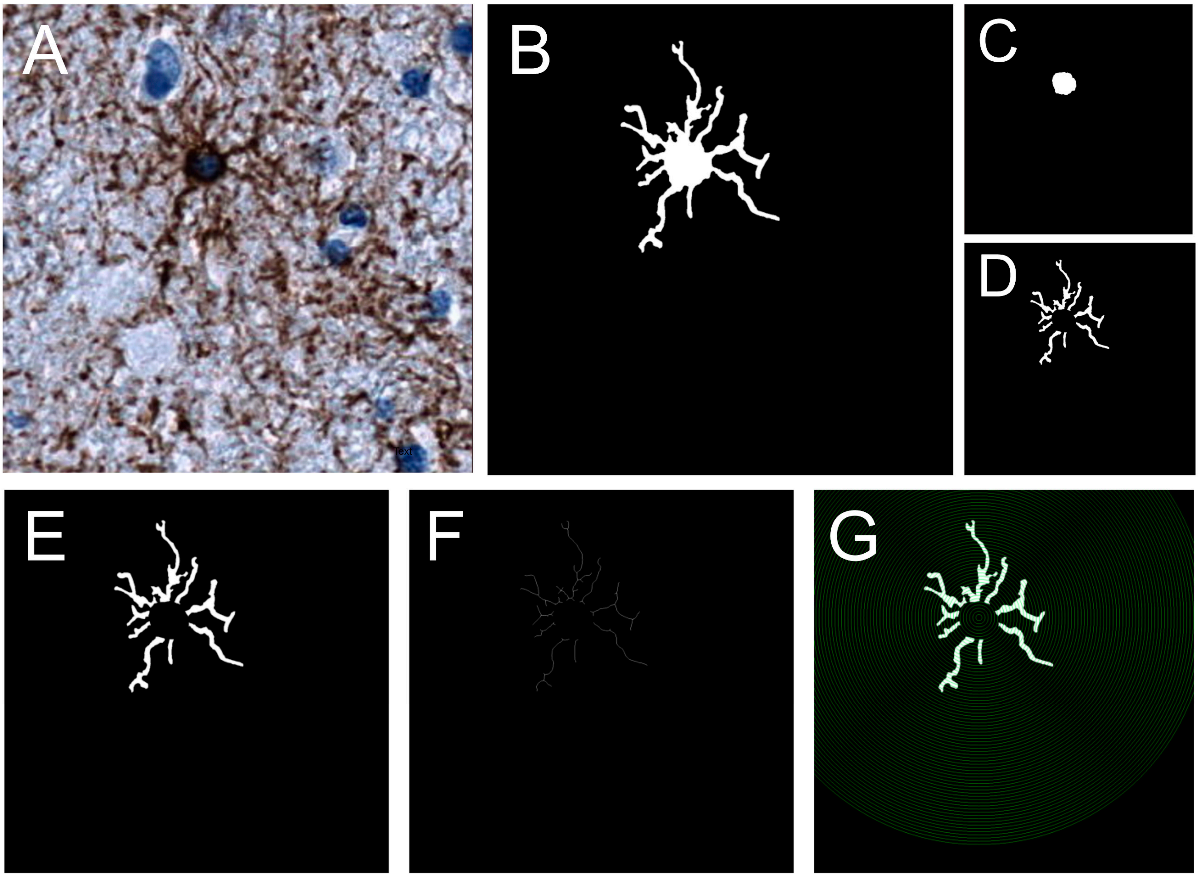

Figure S1. Workflow for astrocyte segmentation and Sholl analysis. (**A**) Original GFAP-immunostained astrocyte image extracted from a whole-slide image, original magnification × 400. (**B**) Binary mask generated in FIJI/ImageJ using intensity thresholding and watershed-based separation. (C) Soma mask used for soma size measurement. (**D**) GFAP-positive process mask after removal of the soma from the Sholl pipeline. (**E**) Processed binary input used for morphometric analysis. (**F**) Skeletonized representation of the astrocytic arbor used for intersection quantification. (**G**) Concentric Sholl rings overlaid for visualization; intersection counts were calculated on the skeletonized image rather than on the overlaid image.
